# Evidence-based teaching: The examination and use of psychological theories in medical education research using the example of cognitive load theory

**DOI:** 10.3205/zma001805

**Published:** 2026-01-15

**Authors:** Hiltraut Paridon

**Affiliations:** 1 Hochschule Fresenius, Dresden, Germany

**Keywords:** Cognitive Load Theory, teaching and learning research, psychology, medical studies

## Abstract

One goal of medical education research is to optimally design teaching and learning processes so that students are supported in acquiring sustainable knowledge and competencies. To this end, education research systematically deals with the conditions and effects of teaching and learning. Therefore, it is situated in the field of teaching and learning research, which is clearly empirically oriented (and not pedagogically-humanistic). This article illuminates the role of empirically tested theories in medical education research using the example of cognitive load theory (CLT). For this purpose, some fundamental aspects of psychological research are discussed first. This is followed by a presentation of CLT, which is based on findings from memory psychology. Based on findings on the modality effect and the split-attention effect, it is illustrated how findings from tested theories can contribute to the development of teaching and learning methods and materials in practice and what limitations exist. Finally, some further developments of CLT and its relevance for medical professionals are presented.

## Abstract (de)

Ein Ziel medizinischer Ausbildungsforschung besteht darin, Lehr- und Lernprozesse optimal zu gestalten, so dass die Studierenden unterstützt werden, nachhaltig Wissen und Kompetenzen zu erwerben. Hierzu beschäftigt sich die Ausbildungsforschung systematisch mit den Bedingungen und Effekten von Lehren und Lernen. Sie ist somit im Bereich der Lehr-Lernforschung angesiedelt, die eindeutig empirisch geprägt ist (und nicht etwa pädagogisch-geisteswissenschaftlich). In dem vorliegenden Artikel wird die Rolle empirisch geprüfter Theorien in der Medizinischen Ausbildungsforschung am Beispiel der Theorie der kognitiven Belastung (Cognitive Load Theory, CLT) beleuchtet. Hierzu werden zunächst einige grundlegende Aspekte der psychologischen Forschung erörtert. Es folgt die Darstellung der CLT, die auf Erkenntnissen der Gedächtnispsychologie basiert. Anhand von Erkenntnissen zum Modalitätseffekt und zum Effekt der geteilten Aufmerksamkeit wird verdeutlicht, wie Erkenntnisse aus geprüften Theorien zur Entwicklung von Lehr-/Lernmethoden und -materialien in der Praxis beitragen können und welche Grenzen hierbei existieren. Abschließend werden einige Weiterentwicklungen der CLT sowie ihre Relevanz für Mediziner*innen dargestellt.

## 1. Introduction

Teaching and learning research addresses, among other things, the question of how teaching and learning processes can be designed to be as sustainable as possible. Psychological research plays a major role here. Since the 19^th^ century, scientific psychology has been strongly natural science-oriented in its research methods, i.e., empirically or experimentally oriented [1]. Psychology is the scientific study of mind and behavior, i.e. how the human mind works and how it influences behavior [2]. Its task is to describe, explain, predict, and influence experience and behavior [3]. It considers information processing, emotions, and behavior in its broadest sense, so that, for example, physiological processes are also investigated [4]. Important theories for teaching and learning research include, for example, Cognitive Load Theory (CLT) [5], [6], [7], theories of metacognition and self-regulation [8], and information processing theory [9]. These theories help to describe and explain learning processes. Based on those theories, measures can be derived that teachers and learners can use to improve the acquisition of knowledge and competencies.

This purpose of the article is twofold. First, it aims to illustrate how empirical psychological research arrives at well-founded scientific insights. In teaching and learning research, this often involves the question of which factors influence learning success and how it can be improved. Second, specific research results will be presented using CLT as a selected theory. Since medical students must understand and retain numerous complex learning contents sustainably, corresponding insights are of high relevance. CLT is one of the evidence-based theories from which practical recommendations for teaching and learning can be derived, which can then in turn be empirically tested in education research.

### 1.1. Study designs in scientific psychology

In scientific psychology – as in medicine – high-quality studies are required that can prove causal relationships. While in medicine, randomized controlled trials (RCTs) are discussed, in psychology, the term “experiment” is generally used [2]. This study design is also postulated by various scientists for pedagogical questions [10], [11]. However, there are also other positions, so that in the German-speaking pedagogical field, a rather liberal methodological pluralism has developed regarding the concept of evidence, which is critically discussed [12]. A study design that is more common in psychological-pedagogical research than in medicine is the “quasi-experiment”. Here, as in the experiment, there is a control group – however, there is no random assignment of participants to the conditions. This is the case, for example, when two groups of learners (e.g., two school classes) already exist and one group receives an intervention, while the other group serves as a control group [13].

### 1.2. Causal research based on theories

Since information processing activities and emotional processes are not directly accessible to observation, the corresponding mental and affective processes must be inferred from behavior [2]. In the pedagogical field, for example, the question is why a student passes an exam well. Causes can be that the person has learned persistently due to high motivation and self-efficacy [14], [15] and has also used effective learning strategies [16]. To find out the relevant reasons (which in turn can be the basis for a behavior change in less successful students), studies are conducted that are based on previously developed theories. As many (human) behaviors are multifactorially determined, different theories are needed that allow assumptions about what behavior different people display in different situations.

### 1.3. Scientific theories and empirical testing

A scientific theory is understood as a complex and ordered system of assumptions that can explain relationships. It is an explanatory model that allows predictions, whereby the model is generalized – as it is not about individual case studies – and reduced – since not all influencing factors can be checked [17]. Predictions that can be derived are formulated as hypotheses, which in turn can then be confirmed or rejected with empirical studies. This includes, for example, the assumption that people with high self-efficacy pursue a goal with greater persistence than people with low self-efficacy [15]. Certain demands are placed on scientific theories. To be empirically revisable, hypotheses must be formulated so that they have the potential to be proven incorrect (falsification principle). If this is not possible, it is a non-scientific theory [2]. The principle of falsification is essential for the empirical sciences, as they focus on disproving theoretical predictions rather than confirming them [18].

### 1.4. Psychology and neuroscience

Psychological theories and the empirical testing of hypotheses remain indispensable despite the significant developments in neuroscience. Neurosciences contribute to the understanding of brain processes and can, for example, help to better explain cognitive performance disorders, but they do not provide information on how information should be prepared so that it can be processed and stored in memory more effectively. The distinction between the physically existing brain and the constructs relevant for mental and emotional processes (such as intelligence, self-efficacy, motivation), which have no physical counterpart, remains of great importance [19].

## 2. The Cognitive Load Theory as an example of a pedagogical-psychological theory

The Cognitive Load Theory (CLT) by Sweller and colleagues [5], [6], [7] is a well-known theory of knowledge acquisition [20]. It provides a basis for numerous empirical studies in various subject areas, such as learning with media, learning from worked examples, and skill acquisition [21], [22]. CLT primarily deals with the learning of complex cognitive content. Learners are often overwhelmed by the number of information elements and their interrelationships that must be processed simultaneously. This impedes meaningful learning. How instructional design can help reduce this (too) high load to enable meaningful learning in complex cognitive domains has become the focus of CLT. The theory assumes that learning occurs best under conditions that are consistent with human cognitive architecture. In doing so, working memory is in the focus of its consideration [23].

### 2.1. Basic memory models in psychology

The study of human memory experienced a great boost with the so-called “cognitive revolution”. An important advancement was the theoretical development of short-term memory [24]. Although this theory has been considered outdated or disproven for quite some time, it can still be found in (non-psychological) textbooks. As an advancement, the theory of working memory was developed, which is also capacity-limited but represents an actively working memory system [25]. Working memory contains a so-called “phonological loop” for verbal material and a “visuospatial sketchpad” for visual material [26]. The loop and the sketchpad serve as subsidiary systems to keep information available, and their use is controlled by a central executive. In addition, information from long-term memory (LTM) is used to solve problems or compare information. Information can also enter LTM without remaining in working memory [25]. Different types of storage and knowledge are distinguished in LTM. Particularly important for CLT is semantic memory, which contains facts, concepts, principles, and rules [27]. According to theory, the contents of semantic memory are stored in a hierarchical network structure and in schemata. Schemata are superordinate mental knowledge structures that contain information about objects, situations, and content in an abstract, generalized form and enable understanding. After intensive practice, they can become automated (e.g., reading) and therefore relieve working memory, so that cognitive capacities are available for other functions [23], [25].

### 2.2. Types of cognitive load in working memory

CLT assumes that every learning process poses a cognitive load on working memory [28]. Since the capacity of working memory is limited, the total cognitive load should ideally not exceed this capacity, as this would negatively affect the learning process. The magnitude of cognitive load during learning depends on both the complexity of the learning content and the design of the learning materials. The theory distinguishes three different types of cognitive load [29].

#### 2.2.1. Intrinsic cognitive load

Intrinsic cognitive load (ICL) results from the inherent complexity of the content. It depends on what is called element interactivity. This refers to the question of how many elements, i.e., knowledge contents, must be processed simultaneously in working memory to understand a learning content, because individual elements can only be understood in relation to other elements. If a learning task can be managed serially, the intrinsic cognitive load is rather low – this is the case, for example, when vocabulary or technical terms need to be learned. The ICL increases when different pieces of information must be actively held in working memory simultaneously, such as when making a medical diagnosis. The learner’s prior knowledge is crucial for content-related cognitive load. If they have extensive prior knowledge and thus schemata are already available in long-term memory, the intrinsic cognitive load decreases [6], [7].

#### 2.2.2. Extraneous cognitive load

Extraneous (also: irrelevant) cognitive load (ECL) depends on the learning environment, the external characteristics of the learning material, and the general conditions during learning. These characteristics are not directly related to the processes of knowledge construction and can even disrupt them if poorly designed [6], [7]. This is the case, for example, when related information in a figure, such as an image and text, is presented spatially separated from each other. Knowledge acquisition is impaired in many situations because the extraneous load is too high. Its proportion of the total cognitive load should be as low as possible [20].

#### 2.2.3. Germane cognitive load

Germane cognitive load (GCL) is the load required for learning and should constitute the essential part of the total cognitive load. It is thus caused by the actual understanding-based learning and contributes to learning success. It is necessary for the construction and automation of schemata in long-term memory and refers to the mental resources expended for this purpose [30]. The greater the engagement in learning-promoting cognitive processes, the higher this type of load and the better the learning or comprehension performance [6], [7]. This load can be promoted with examples and exercises that use different contexts, thus leading to more robust mental models than examples or exercises that use similar contexts [29].

### 2.3. Total working memory load

**Figure 1:**
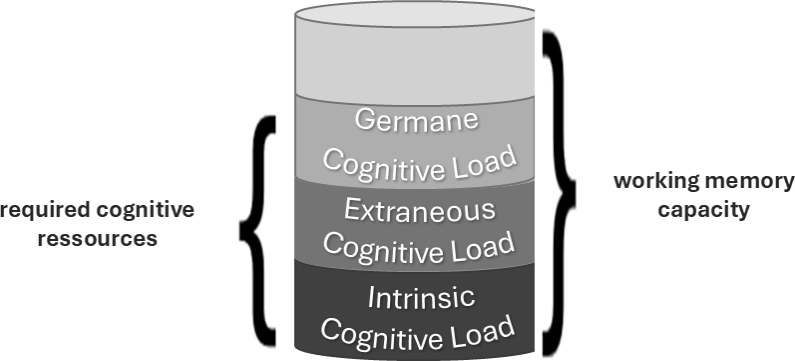
Schematic illustration of the cognitive load theory (own representation on the basis of [51])

The three types of loads sum up to a total cognitive load that strains working memory. If the total load is too high, learning becomes less effective. By managing the intrinsic load and, above all, by reducing the extraneous load, working memory can be relieved, so that more resources are available for germane cognitive load and a greater progress in learning is possible. Figure 1 (Fig. 1) shows the three types of cognitive load and their relationship to working memory.

### 2.4. Studies on the modality effect and the split-attention effect

As described above, hypotheses are derived from theories and then empirically tested. Of the numerous hypotheses derivable from CLT, two frequently confirmed assumptions will be presented as examples.

#### 2.4.1. Modality effect

The “modality effect” describes the phenomenon that complex visual displays are better understood when the explanatory words are presented in a different modality, i.e. auditory text plus visual representation). Since working memory has different processing mechanisms for visual and auditory information, the use of two modalities allows for more efficient use of limited resources than one modality alone. In one experiment, a group of learners was presented with information on interpreting line plots purely visually, and in the other group, in two modalities [31]. The results showed that the audiovisual presentation was superior to the purely visual one. Crucial for this finding was that both sources of information were connected and essential for the comprehensibility of the material. In experiments with geometric problems, the superiority of two modalities was also demonstrated [32]. In three experiments from the field of electrical engineering, it was also shown that participants who used audiovisual materials achieved better results than participants who used a purely visual format [33]. However, these results were only achieved with instructions containing high intellectual content. A meta-analysis on the modality effect concludes that learners across different learning materials and age groups performed better when materials consisted of graphics and spoken text, compared to graphics with printed text [34]. However, the results depend on the degree of element interactivity and the presentation speed. With higher element interactivity (and thus complexity of the material), the modality effect is stronger (d=0.72). Furthermore, the modality effect is greater when the presentation duration is predetermined by the system compared to self-determined duration and with shorter compared to longer texts [34].

#### 2.4.2. Effect of split attention

The “split-attention effect” states that a spatially separated presentation of related (visual) information, such as an image and its corresponding text, leads to poorer learning performance. The information should be presented spatially together, so that, for example, labels in an illustration are near the corresponding image elements [6]. The separated presentation leads to increased extraneous load [35]. A meta-analysis showed that integrated designs improve learning across many moderator variables with an overall effect size of g=0.63 [36].

### 2.5. Further studies and developments of CLT

The assumptions derived from CLT have primarily been proven for learners with low prior knowledge, but not for learners with high prior knowledge. This expertise reversal effect is found in both the split-attention effect and the modality effect [6], [37], [38]. Experimental studies have shown that the use of two modalities led to higher learning effects in novices, but this effect disappeared or even reversed with increasing expertise [39]. Furthermore, it has been shown that a so-called redundancy effect can occur when using two modalities. This effect leads to a deterioration in learning performance if the content of the auditory material is redundant to the visual material. The redundancy effect becomes stronger when the information has a high complexity [40].

In addition to the differentiation of principles that have emerged from further research, CLT has led to the development of other theories, such as Mayer’s “cognitive theory of multimedia learning” [41], [42]. The “Cambridge handbook of multimedia learning” presents numerous findings and prepares them for practical application. The superiority of the design principles presented there has also been demonstrated in medical students [43].

Further developments relate to the foundation of CLT based on findings from evolutionary psychology and the development of new instruments to measure the different types of cognitive load [7], [44]. In addition, new constructs have been incorporated into the theory. These include working memory exhaustion and the effect of affective factors [45]. Studies show that working memory resources can be exhausted, leading to a decrease in performance. Resources can be restored through a break [46]. Affective factors such as emotions, stress, and uncertainty can not only increase extrinsic cognitive load, but also intrinsic load since affective factors are an element of many real professional situations where complex skills are required [45].

### 2.6. Design recommendations for medical education

To specify CLT for medical education, fifteen recommendations were discussed in an article in the journal “Medical Education” and each illustrated with an example, with which external load can be reduced, intrinsic load can be controlled, and germane load can be optimized [6]. A more recent article describes further applications of CLT in various areas of medical education [47]. Some examples will be mentioned here to clarify recommendations derived from theory.

- The split-attention principle states that multiple spatially or temporally distributed information sources should be replaced by an integrated information source. From this, it can be deduced that it is better to give students instructions for operating a medical device exactly when they are using the device, rather than informing them in advance. Furthermore, it can be deduced that it is more advantageous to distribute lecture slides beforehand so that students can focus their attention on making connections between the presented content and their prior knowledge, instead of copying the projected slides.
- When a visual computer animation is shown, e.g., the functioning of the lungs or the digestive tract, it can be deduced from the modality effect that it is better to supplement it with an oral explanation than a written explanation on the screen.
- According to the completion principle, it is better to provide pre-made tasks that only need to be partially completed. For example, if students are supposed to calculate the sensitivity and specificity of a diagnostic test, they can be given a worksheet with tasks already partially filled in. In an operation, medical students should initially begin by taking over parts of the operation and observing the rest, instead of having to perform it completely alone immediately.
- Similar to the completion principle is the worked-example effect. Learners should be given an exemplary solution to a problem so that they can orient themselves to it and criticize it – but not have to solve the problem on their own. For inexperienced students, for example, it is better to let them criticize a pre-made treatment plan instead of having them create such a plan themselves. However, there is an important exception to this – this refers to the expertise reversal effect. As learners’ competence increases, the effect of worked examples becomes less effective and eventually superfluous or even counterproductive for learning outcomes.
- According to the variability effect, higher variability in different learning exercises leads to better transfer performance. For this reason, learning tasks should vary in all dimensions in which they also vary in reality – i.e., outside the learning situation. For example, if clinical symptoms of a disease are described, they should be illustrated using patients of different genders, ages, medical histories, etc.

### 2.7. Further developments of CLT in medical education

As described in Section 2.5, CLT has been further developed in various areas. This also includes the development of expertise over time. In a 2021 article, the authors present a model that specifically considers this aspect in clinical practice [45]. In this recontextualization of CLT for complex professional areas such as medicine – in addition to the exhaustion of working memory and the consideration of emotions – the construction of schemas and automation are essential. By developing schemas in LTM and automating them, the load on working memory is reduced.

Experienced medical professionals develop strategies that enable them to reduce intrinsic cognitive load by constructing and automating mental schemas. This allows them to better cope with the complexity of medical cases as well as with emotions, stress, and uncertainty that their work may entail. Experienced medical professionals can also reduce their extraneous cognitive load by knowing which information in a situation is less relevant and therefore negligible. This releases capacity in working memory. This extended model needs to be verified in further studies.

Studies on other topics in medical education, such as context specificity, also show close connections to CLT. The fluctuations observed in performance during clinical decision-making (clinical reasoning) could be explained with the help of CLT, as context specificity is closely related to working memory load [48], [49].

## 3. Conclusion

Prospective medical professionals are confronted with an extraordinary amount of complex learning content that they must retain sustainably to be able to retrieve the necessary (action-oriented) knowledge in a given situation. Learners can be supported through the application of evidence-based teaching and learning strategies. This requires studies with high external evidence [50] that test theories also regarding their practical applicability. Cognitive Load Theory has initiated intensive research activities in the field of teaching and learning research. Numerous studies have dealt with different assumptions arising from CLT. It enables predictions on how teaching and learning processes can be designed to improve learning success. Many studies refer to the design of media, which also includes the use of presentations in lectures and seminars. The research results and the design recommendations derived from them can be relatively easily implemented by teachers, thus supporting students' learning success. A key goal in applying CLT is to relieve working memory by reducing extraneous cognitive load as much as possible, thereby making more resources available for germane load. However, it should be noted that the exact circumstances of the learning situation should be considered, as negative consequences may arise otherwise [39].

In addition to CLT, other theories – such as the ones on metacognition, self-regulation, and information processing mentioned in the introduction [8], [9] – can help to make learning processes more sustainable. Medical education research can provide information on how research findings can be specifically implemented in the training of future medical professionals.

## Author’s ORCID

Hiltraut Paridon: [0000-0002-8652-7350]

## Competing interests

The author declares that she has no competing interests.

## References

[1] Lück HE, Guski-Leinwand S (2004). Geschichte der Psychologie.

[2] Myers DG (2014). Psychologie.

[3] Wolstein J, Schütz A, Lautenbacher S, Schütz A, Brand M, Selg H, Lautenbacher S (2015). Das Studium der Psychologie und Berufsperspektiven.

[4] Prinz, W, Müsseler J, Rieger M, Müsseler J, Rieger M (2017). Einleitung – Psychologie als Wissenschaft.

[5] Sweller J (2010). Element interactivity and intrinsic, extraneous, and germane cognitive load. Educ Psychol Rev.

[6] van Merriënboer JJ, Sweller J (2010). Cognitive load theory in health professional education: design principles and strategies. Med Educ.

[7] Sweller J, van Merriënboer JJ, Paas F (2019). Cognitive Architecture and Instructional Design: 20 Years Later. Educ Psychol Rev.

[8] Perels F, Dörrenbächer-Ulrich L, Landmann M, Otto B, Schnick-Vollmer K, Schmitz B, Wild E, Möller J (2021). Selbstregulation und selbstreguliertes Lernen.

[9] Gruber H, Stamouli E, Wild E, Möller J (2021). Intelligenz und Vorwissen.

[10] Goldacre B (2013). Building evidence into education.

[11] Blakemore SJ (2018). Das Teenager-Gehirn.

[12] Stark R (2017). Probleme evidenzbasierter bzw. -orientierter pädagogischer Praxis. Z Päd Psychol.

[13] Rost D (2022). Interpretation und Bewertung pädagogischer und psychologischer Studien.

[14] Deci ED, Ryan RM (1993). Die Selbstbestimmungstheorie der Motivation und ihre Bedeutung für die Pädagogik. Z Pädagogik.

[15] Schwarzer R, Jerusalem M, Jerusalem M, Hopf, D (2002). Matthias: Das Konzept der Selbstwirksamkeit.

[16] Imhof M (2016). Psychologie für Lehramtsstudierende.

[17] Digitales Wörterbuch der deutschen Sprache. Theorie.

[18] Greve W, Thomsen T (2019). Entwicklungspsychologie: Eine Einführung in die Erklärung menschlicher Entwicklung.

[19] Schumacher R, Stern E (2012). Neurowissenschaften und Lehr-Lern-Forschung: Welches Wissen trägt zu lernwirksamem Unterricht bei?. Dtsch Schule.

[20] Wild E, Möller J (2020). Pädagogische Psychologie.

[21] Plass JL, Moreno R, Brünken R (2010). Cognitive load theory.

[22] Sweller J, Ayres P, Kalyuga S (2011). Cognitive Load Theory.

[23] Paas F, Renkl A, Sweller J (2004). Cognitive Load Theory: Instructional Implications of the Interaction between Information Structures and Cognitive Architecture Instructional Science. Instruct Sci.

[24] Atkinson RC, Shiffrin RM, Spence K, Spence J (1968). Human memory: A proposed system and ist control processes.

[25] Baddeley A (1996). Exploring the Central Executive. Quart J Ex Psycho.

[26] Stefan B (2015). Arbeitsgedächtnis: Vergangenheit, Gegenwart und Zukunft eines theoretischen Konstrukts. Psychol Rundschau.

[27] Hasselhorn M, Gold A (2022). Pädagogische Psychologie.

[28] Sweller J (1994). Cognitive load theory, learning difficulty, and instructional design. Learn Instruct.

[29] Clark RC, Nguyen F, Sweller J (2006). Efficiency in learning: Evidence-based guidelines to manage cognitive load.

[30] Debue N, van de Leemput C (2014). What does germane load mean? An empirical contribution to the cognitive load theory. Front Psychol.

[31] Leahy W, Chandler P, Sweller J (2003). When auditory presentation should and should not be a component of multimedia instruction. Appl Cogn Psychol.

[32] Mousavi SY, Low R, Sweller J (1995). Reducing cognitive load by mixing auditory and visual presentation modes. J Educ Psychol.

[33] Tindall-Ford S, Chandler P, Sweller J (1997). When two sensory modes are better than one. J Ex Psychol.

[34] Ginns P (2005). Meta-analysis of the modality effect. Learn Instruct.

[35] Pouw W, Rop G, de Koning B, Paas F (2019). The cognitive basis for the split-attention effect. J Exp Psychol Gen.

[36] Schroeder NL, Cenkci, AT (2018). Spatial Contiguity and Spatial Split-Attention Effects in Multimedia Learning Environments: a Meta-Analysis. Educ Psychol Rev.

[37] Kalyuga S, Chandler P, Sweller J (1998). Levels of expertise and instructional design. Hum Factors.

[38] Tabbers HK, Martens RL, van Merriënboer JJ (2004). Multimedia instructions and cognitive load theory: Effects of modality and cueing. Br J Educ Psychol.

[39] Kalyuga S, Chandler P, Sweller J (2000). Incorporating learner experience into the design of multimedia instruction. J Educ Psychol.

[40] Reinwein, J (2012). Does the modality effect exist? And if so, which modality effect?. J Psycholinguist Res.

[41] Mayer RE, Mayer RE (2005). Cognitive Theory of Multimedia Learning.

[42] Mayer RE, Fiorella L (2022). The Cambridge Handbook of Multimedia Learning.

[43] Issa N, Schuller M, Santacaterina S, Shapiro M, Wang E, Mayer RE, DaRosa DA (2011). Applying multimedia design principles enhances learning in medical education. Med Educ.

[44] Sweller J (2024). Cognitive load theory and individual differences. Learn Individ Diff.

[45] Szulewski A, Howes D, van Merriënboer JJG, Sweller J (2021). From Theory to Practice: The Application of Cognitive Load Theory to the Practice of Medicine. Acad Med.

[46] Leahy W, Sweller J (2019). Cognitive load theory, resource depletion and the delayed testing effect. Educ Psychol Rev.

[47] Young JQ, van Merriënboer JJ, Durning S, ten Cate O (2014). Cognitive Load Theory: Implications for medical education: AMEE-Guide, 2014, No. 86. Med Teach.

[48] Durning SJ, Artino AR, Boulet JR, Dorrance K, van der Vleuten C, Schuwirth L (2012). The impact of selected contextual factors on experts' clinical reasoning performance (does context impact clinical reasoning performance in experts?). Adv Health Sci Educ.

[49] Konopasky A, Artino AR, Battista A, Ohmer M, Hemmer PA, Torre D, Ramani D, van Merriënboer JJ, Teunissen PW, McBee E, Ratcliffe T, Durning SJ (2020). Understanding context specificity: the effect of contextual factors on clinical reasoning. Diagnosis (Berl).

[50] Mörth M, Paridon H, Enders N, Ulrich I, Rhein R, Wildt J (2003). Psychologie als eine Grundlage der Hochschuldidaktik. Ansatz für eine interdisziplinäre Annäherung.

[51] Krieglstein F, Beege M, Rey GD, Ginns P, Krell M, Schneider S (2022). A Systematic Meta‑analysis of the Reliability and Validity of Subjective Cognitive Load Questionnaires in Experimental Multimedia Learning Research. Educ Psychol Rev.

